# New Approach Using the Real-Time PCR Method for Estimation of the Toxic Marine Dinoflagellate *Ostreopsis* cf. *ovata* in Marine Environment

**DOI:** 10.1371/journal.pone.0017699

**Published:** 2011-03-03

**Authors:** Federico Perini, Anna Casabianca, Cecilia Battocchi, Stefano Accoroni, Cecilia Totti, Antonella Penna

**Affiliations:** 1 Department of Biomolecular Sciences, University of Urbino, Pesaro, Italy; 2 Department of Marine Sciences, Polytechnic University of Marche, Ancona, Italy; East Carolina University, United States of America

## Abstract

**Background:**

We describe the development and validation of a new quantitative real time PCR (qrt-PCR) method for the enumeration of the toxic benthic dinoflagellate *Ostreopsis* cf. *ovata* in marine environment. The benthic *Ostreopsis* sp. has a world-wide distribution and is associated during high biomass proliferation with the production of potent palytoxin-like compounds affecting human health and environment. Species-specific identification, which is relevant for the complex of different toxins production, by traditional methods of microscopy is difficult due to the high morphological variability, and thus different morphotypes can be easily misinterpreted.

**Methodology/Findings:**

The method is based on the SYBR I Green real-time PCR technology and combines the use of a plasmid standard curve with a “gold standard” created with pooled crude extracts from environmental samples collected during a bloom event of *Ostreopsis* cf. *ovata* in the Mediterranean Sea. Based on their similar PCR efficiencies (95% and 98%, respectively), the exact rDNA copy number per cell was obtained in cultured and environmental samples. Cell lysates were used as the templates to obtain total recovery of DNA. The analytical sensitivity of the PCR was set at two rDNA copy number and 8.0×10^−4^ cell per reaction for plasmid and gold standards, respectively; the sensitivity of the assay was of cells g^−1^ fw or 1^−1^ in macrophyte and seawater samples, respectively. The reproducibility was determined on the total linear quantification range of both curves confirming the accuracy of the technical set-up in the complete ranges of quantification over time.

**Conclusions/Significance:**

We developed a qrt-PCR assay specific, robust and high sample throughput for the absolute quantification of the toxic dinoflagellate *Ostreopsis* cf. *ovata* in the environmental samples. This molecular approach may be considered alternative to traditional microscopy and applied for the monitoring of benthic toxic microalgal species in the marine ecosystems.

## Introduction

The qrt-PCR is a powerful technique able of accurate and sensitive estimation of microbial species abundance in different environments for applied ecology studies. Several qrt-PCR assays, mostly based on SYBR Green I, Taqman and Molecular Beacon technologies, have been developed for a large number of toxic microalgal species quantification [1], [2], [3]. The method used in these studies is to generate a standard curve using plasmids containing target ribosomal DNA sequences, or genomic DNA extracted from cultured cells or resting stages with known concentrations of the target microbial species. Although ribosomal genes have been the target molecules of choice in the development of the qrt-PCR assays, few of the data reported so far in the literature show that in some microbial taxa the rRNA genes are present as pseudogenes and also organised in extra-chromosomal molecules [4], [5]. Given this potential variation in the rRNA gene copy number [6], the strategy of pooling different genomic DNAs derived from several cultured isolates of the same species to generate a standard for quantifying environmental samples may affect the results of the qrt-PCR assay [7]. Moreover, other qrt-PCR assays applied to environmental samples have yielded highly variable results. This may be due to other factors, such as different amplification efficiencies in standard and field samples, and low and unreliable recovery of total DNA extracted using conventional methods [8].

The toxic genus *Ostreopsis* includes various benthic species which have world-wide distribution from tropical to temperate coastal sites and are associated with the production of potent palytoxin-like (PLTX) compounds [9]. In tropical benthic assemblages, *Ostreopsis* spp. co-occurs with other harmful benthic dinoflagellates, including *Gambierdiscus* spp., responsible for ciguatera [10]. In last decade, *Ostreopsis* spp. blooms regularly occurred in the warm temperate coasts of the Mediterranean Sea [11], [12], [13] and have recently been associated both with human poisoning by toxic aerosols [14], [15] and with mortality of benthic organisms caused by water deterioration or by direct toxin intake through the food web [16]. Taxonomy of the *Ostreopsis* species based only on morphological characteristics is rather controversial due to the high morphological variability of both natural populations and cultured specimens [17] and therefore, species-specific identification by traditional method of microscopy is extremely difficult. With respect to the Mediterranean Sea, two *Ostreopsis* species, previously characterised as *O.* cf. *ovata* and *O.* cf. *siamensis* by both morphological and genetic analyses, have been found together in bloom events in several coastal areas [18], [19]. Of these, the *O.* cf. *ovata* genotype seems to predominate and has been found with greater frequency and abundance in all analysed samples from the Mediterranean and the rest of the world [20]. Correctly identifying and quantifying these two *Ostreopsis* species simultaneously is therefore crucial, given that distinct species can produce different toxic compounds with a variety of potential risks to public health, the environment and the economic activities of tourism and aquaculture [21].

In this study, we describe the development of the first method based on qrt-PCR for species-specific identification and enumeration of *Ostreopsis* cf. *ovata* from macroalgae and surface seawater samples, which takes into account all the above-mentioned qrt-PCR problems and biases. The species-specific primers designed in the LSU (Large Subunit) rRNA gene were first validated in different *O.* cf. *ovata* isolates and then in field samples by qrt-PCR. The new element in this assay is the construction of a standard curve using crude lysates of pooled samples collected during a *O.* cf. *ovata* bloom. We demonstrated that this standard has the same amplification efficiency of the generated plasmid standard containing the target LSU gene. By the data comparison of the two curves we are able to calculate the cell number and LSU gene copy number per cell of the *O.* cf. *ovata* in the bloom. The qrt-PCR was also compared with the traditional microscopy analysis.

This new method proved to be a more accurate and specific alternative molecular technique to microscopy for investigating the population dynamics of benthic microbial species.

## Materials and Methods

### Microalgal cultures

*Ostreopsis* cf. *ovata* isolates were obtained from Italian coastal waters (Mediterranean Sea) during the summers of 2008 and 2009 and are listed as follows: *O.* cf. *ovata* CBA165 (Pisa, Tyrrhenian Sea), CBA166 (Trieste, northern Adriatic Sea), CBA1273 (Genoa, northern Tyrrhenian Sea), CBA1298 (Livorno, Tyrrhenian Sea), CBA1377 (Bari, southern Adriatic Sea), CBA1346 (Conero Riviera, central Adriatic Sea). The cultures were maintained in F/4 medium at 23±1°C. Light was provided by cool-white fluorescent bulbs (photon flux of 100 µE m^−2^ s^−1^) on a standard 14∶10 h light-dark cycle. Culture sub-samples were fixed with Lugol's iodine solution and stored at +4°C until molecular analysis. *O.* cf. *siamensis* CNRT5 (Taormina, Ionian Sea) was used as a control in the species-specific qrt-PCR experiments.

### Environmental samples

A total of 43 samples of the green macroalga *Ulva rigida* and of surface seawater were collected at Portonovo (Conero Riviera) during the period March–November 2009. Samples of *U. rigida* were harvested at a depth of 20–40 cm and treated according to [13]. Seawater samples (50 ml) were collected in triplicates using polyethylene bottles. The macroalgal wash seawater and surface seawater samples, were fixed with neutralized formalin (0.8% final concentration) and stored at +4°C until molecular analysis.

### Microscopy analyses

Subsamples of the macroalgal wash water (10 ml) and the surface seawater (25–40 ml) samples were settled for 25–40 h in Utermöhl chambers. *Ostreopsis* spp. were counted on the entire sedimentation chamber under an inverted microscope (Axiovert 40 CFL and Axiovert 135H, Zeiss or a Leitz DM-II) at 200 or 400× magnification. Abundances in macroalga and surface seawater samples were expressed as number of cells per gram of fresh weight (cells g^−1^ fw) and number of cells per liter (cells l^−1^), respectively. *Ostreopsis* spp. were identified under epifluorescence microscope after samples were treated with Calcofluor, and Scanning Electron Microscopy according to [22].

### Lysis of environmental and culture samples

The final volume of processed environmental sub-samples (macrophytes and surface seawater), obtained as described above, was 40 ml. Culture and field sub-samples were concentrated by centrifugation at 4000×g for 15 min at room temperature. Cell pellets were carefully washed with 1 ml sterile artificial seawater, centrifuged at 7000×g for 15 min, and stored at −80°C or immediately processed. Pellets of cultures and field samples were resuspended respectively in 500 µl and in 250, 500 or 1000 µl of lysis buffer (10 mM Tris-HCl pH 8.3, 50 mM KCl, 0.5% Nonidet P40, 0.5% Tween 20, 2.5 mM CaCl_2_, 0.1 mg ml^−1^ proteinase K). The suspension was sonicated for 10 sec at 50 W with Ultrasonic Homogenizer LABSONIC (B. Braun, Biotech International, Germany) and incubated at 55°C for 3 h by vortexing every 30 min. Finally, the samples were incubated at 100°C for 5 min to inactivate proteinase K. After centrifugation at 12000×g for 1 min at room temperature to precipitate cell debris, the supernatants containing total DNA or crude extracts were transferred into new tubes and diluted at 1∶10 and 1∶100 for the qrt-PCR assay or stored at −80°C and processed within two weeks. The genomic DNA of the *Ostreopsis* cultures contained in the crude extracts was quantified after incubation at 55°C and before boiling using a Qubit fluorometer with a Quant-iT dsDNA HS Assay Kit, as recommended by the manufacturer (Invitrogen, Carlsbard, CA, USA).

### *Ostreopsis* cf. *ovata* primer design

The design of the species-specific primers was based on all *Ostreopsis* and other related dinoflagellate consensus LSU rRNA gene sequences available from GenBank and [20] using OLIGO 6.0 software. The sequence alignment was constructed using CLUSTALX2 [23]. The species-specific primers for amplification of 204 bp (Tm = 81.8°C) of *O.* cf. *ovata* were Ovata rt forward (5′-TTTGATCACTTTGGCAATCT-3′) and Ovata rt reverse (5′-TGAACTTTACCATGCCATTAG-3′). The primers were synthesised by Eurofins MWG operon (Ebersberg, Germany).

### Species-specificity of the qrt-PCR assay

The species-specificity of the primers was examined *in silico* using BLAST and tested in qrt-PCR with purified genomic DNA of *O.* cf. *ovata* and *O.* cf. *siamensis* from cultures. Species-specificity was also assayed with purified genomic DNA from 10 macrophyte samples collected in a coastal area (Pesaro, central Adriatic Sea) where *Ostreopsis* spp. had not been detected by microscopy analysis. Moreover, the potential presence of target extracellular DNA fragments of *O.* cf. *ovata* in the environmental samples (macrophytes and surface seawater) was also checked. A total amount of 40 ml of 10 environmental samples of *Ulva rigida* collected during an *O.* cf. *ovata* bloom was filtered onto filter type TSTP with 3 µm size pores (Millipore, Billerico, MA, USA) to separate cells from the seawater matrix. Aliquot of 2 µl of the flow through was analysed by the qrt-PCR assay before (first qrt-PCR), and after (second qrt-PCR) by centrifugation at 4000×g for 10 min at room temperature.

### Construction of plasmid and standard curves

The 638 bp partial LSU rDNA region was amplified with LSU D1R and LSU D2C primers [24] from purified *O.* cf. *ovata* CBA165 genomic DNA. The fragment was cloned into the pCR 2.1 vector (Invitrogen, Carlsbard, CA, USA) and the derived pLSUO plasmid DNA was purified using Qiaprep Miniprep kit (QIAGEN, Valencia, CA, USA). Plasmid concentration was measured with a Qubit fluorometer following the manufacturer's instructions. Plasmid copy number was calculated using the following formula: molecules µl^−1^ = (A×6.022×10^23^) (660×B)^−1^, where A is the plasmid concentration (g µl^−1^), B is the plasmid length containing the cloned sequence, 6.022×10^23^ is the Avogadro's number and 660 is the average molecular weight of one base pair. The plasmid standard curve for *O.* cf. *ovata* was obtained amplifying a specific internal fragment of 204 bp from 10-fold scalar dilutions with copy number ranging from 10^6^ to 10^2^ (three replicates), and from 10, 5 and 2 molecules (4 replicates) (pLSUO standard curve). A second standard curve (gold standard) was generated by amplifying the 204 bp specific fragment from a mixed *O.* cf. *ovata* crude extract of 2000 cell pool from *U. rigida* samples (n = 4) collected during the bloom event. This calibration curve was generated using selected cell dilutions as illustrated below (see Assay reproducibility in Results section). In all experiments, negative controls (NTC) containing MilliQ water were tested in triplicate.

### Quantitative real-time PCR assays of cultured and field samples

Qrt-PCR of *O.* cf. *ovata* was performed in a final volume of 25 µl containing Hot-Rescue Real Time PCR Kit SG (Diatheva, Fano, Italy) based on double-stranded DNA binding dye SYBR Green I, primers at a final concentration of 300 nM, 0.5 U of Hot-Rescue Taq DNA polymerase, and 2 µl undiluted, 1∶10 and 1∶100 diluted sub-samples of crude extracts. All amplification reactions were carried out using a Step-one Real-time PCR System (Applied Biosystems, Foster City, CA, USA). The thermal cycling conditions consisted of 10 min at 95°C, followed by 40 cycles at 95°C for 15 s and 60°C for 1 min. A gold standard curve was included in each PCR reaction.

### Data analysis

Acquisition of qrt-PCR data and subsequent analyses were carried out using StepOne Software v. 2.1. Because of our assay used SYBR Green I based amplicon detection, a dissociation curve was constructed after the real time PCR to check for primer dimers, contaminating DNA, and PCR products from misannealed primers.

Standard curves were created automatically and accepted when the slopes were between −3.44 and −3.32 (95–100% efficiency) and the minimum value of the correlation coefficient (R^2^) was 0.96. The amplification percentage efficiency was calculated as (10 ^(−1/slope)^−1)×100. The rDNA copy number per cell and number of *O.* cf. *ovata* cells in the environmental samples (macrophyte and seawater) were calculated by interpolation of the Ct (threshold cycle) experimentally determined on pLSUO and gold standard curves, respectively, taking into account the lysis buffer volume and dilution factor of the crude extracts. The LSU rDNA copy and cell numbers of *O.* cf. *ovata* were determined in the environmental samples.

In addition, where there was non-amplification, the samples were further analysed and classified as follows: a) if a qrt-PCR amplification yielded a Ct value<Ct value from 2-copy plasmid and 0.0008 cell of gold standard, the sample was quantifiable and the LSU rDNA and cell numbers were determined (sample n. 20); b) if the Ct value>Ct value derived from 0.0008 cell, the sample was defined as positive for the presence of *O.* cf. *ovata* but below this quantification limit (sample n. 21); c) if a negative amplification was reproduced, samples were checked in the spike-qrtPCR for the presence of PCR inhibitors by adding 2 plasmid copies to 1∶1, 1∶10 and 1∶100 crude extract dilutions. If the Ct values corresponded to those obtained from 2 pLSUO copy number amplification, the samples were not inhibited and these data were reported as not detected (n.d.). *O.* cf. *ovata* abundance on *U. rigida* was normalised to cells g^−1^ fw, while *O.* cf. *ovata* abundance in surface seawater was normalised to cells l^−1^.

Statistical analyses were performed with non-parametric Mann-Whitney, KrusKal-Wallis and Spearman correlation tests with the MedCalc program (MedCalc Software, Mariakerke, Belgium), with a *p*<0.05 determining significance.

## Results

### Optimisation of lysis procedure

In order to check the efficiency of the DNA extraction procedure, 4 samples of cultured *O.* cf. *ovata* CBA165 harvested at 6^th^ day of growth and containing 50000 (a), 20000 (b), 10000 (c) and 5000 (d) cells respectively were lysed with 500 µl of buffer and tested by qrt-PCR assay. The rRNA gene copy numbers per cell (± SD) calculated from these samples (9875±275; 7604±96; 8902±1269; 9517±1621 for samples a, b, c and d, respectively) were not significantly different (*p*>0.05). The results showed that the DNA extraction procedure was not affected by different cell concentrations within the tested range.

### Assay specificity

The species-specificity of the primers designed to target *O.* cf. *ovata* was examined *in-silico* using BLAST and the results indicated that they were highly specific. Species-specificity was also tested by qrt-PCR on crude extracts from macrophyte samples where *Ostreopsis* spp. had not been detected by microscopy. Negative amplifications were obtained. The species-specificity of the primers was also assayed in cultures of *O.* cf. *ovata* and other species of *Ostreopsis*, such as *O.* cf. *siamensis*. Positive amplification only with *O.* cf. *ovata* cultures demonstrated the species-specificity of primers. Furthermore, other several environmental samples containing high background of different microbial taxa assemblages collected in different coastal localities of the Mediterranean Sea in 2008–2009 summers were analysed. The results showed the taxon – specificity signal of the amplification of the mixed microphytobenthic assemblage DNA only in those samples resulted positive for the presence of *O.* cf. *ovata* without any other aspecific amplified product (data not shown). The potential presence of *O.* cf. *ovata* extracellular DNA fragments in two different types of environmental samples, macrophytes and surface seawater (n = 6), was tested by first and second qrt-PCR assay as reported in the Materials and Methods section. The qrt-PCR assays gave Ct value means of 34.56±0.8 (n = 3 macrophyte samples) and 33.4±0.5 (n = 3 seawater samples)>the Ct value obtained with 2-copy plasmid dilutions (32.09±0.71), demonstrating a negligible presence of free *O.* cf. *ovata* DNA.

### PCR inhibitors and spiking experiments

To check for the presence of potential inhibitors, ten-fold serial dilutions of crude extract (1∶1, 1∶10, 1∶100) were tested by qrt-PCR and the amplification efficiency evaluated. To obtain optimal quantification, only the PCR products of the same sample with a Ct difference between 3.3 and 3.4 (ΔCt of 3.3 corresponds to an optimal efficiency of 100%) were accepted. None of the dilutions was eliminated for the quantification assay, as they all fell within this range (data not shown). In addition, the putative presence of PCR inhibitors in *O.* cf. *ovata* samples with negative amplification was verified by adding 2 copies of pLSUO plasmid to the 1∶1, 1∶10 and 1∶100 dilutions of crude extracts of environmental samples. The spiked qrt-PCR amplifications showed that the Ct values of the 2 molecules (31.89±0.45, n = 26) were not significantly different to the 2 molecules of the plasmid standard curve (32.08±0.71, n = 8) (*p*>0.05), indicating that the negative amplification of the environmental samples was in fact due to the absence of LSU rDNA target sequences and not to the presence of inhibitors.

### Standard curves, dynamic range and sensitivity of the assay

The adoption of pLSUO plasmid as standard was validated. The conditions of the environmental samples were simulated by adding 2 µl of crude extract sample, which had been analysed by qrt-PCR to ensure absence of *O.* cf. *ovata* DNA, to all pLSUO scalar dilution samples. The results showed that this standard had the same efficiency as that obtained only with pLSUO scalar dilutions (slopes = 3.50 and 3.44, respectively, Δs<0.01).

**Figure 1:**
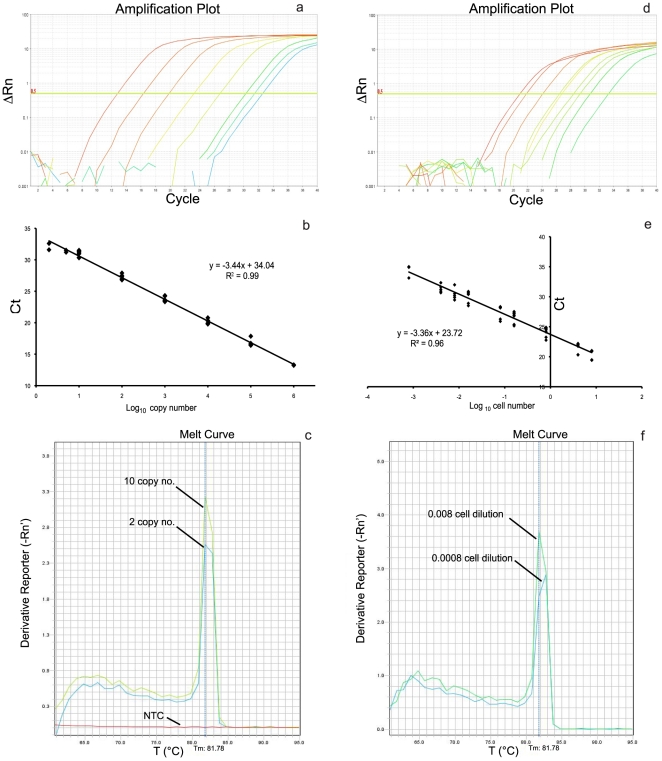
Dynamic range, sensitivity and specificity of the qrt-PCR assay. A typical amplification plot (a, d) and the corresponding standard curve (b, e) for the pLSUO and gold standards are shown, respectively. A 204 bp fragment of the LSU gene were amplified in a 10^6^, 10^5^, 10^4^, 10^3^, 10^2^, 10, 5 and 2 copy dilutions of the pLSUO plasmid (a) and in a 8, 4, 0.8, 0.16, 0.08, 0.016, 0.008, 0.004 and 0.0008 *O.* cf. *ovata* cell dilutions (d) of the lysed pool from macroalgae samples. The fluorescence intensity (ΔRn) expressed as logarithmic scale is plotted vs cycle number. Only one replicate is shown. The mean standard curve is obtained by the correlation of Ct values and log_10_ input plasmid copy (b) or environmental *O.* cf. *ovata* cell number (e) measured in 8 independent experiments ± SD, respectively. Melting curve of the the 204 bp amplified fragments of the pLSUO plasmid (c) and environmental *O.* cf. *ovata* cells (f) generated by qrt-PCR. To better display the melting curves, only the low input pLSUO plasmid copy number (10, 2 and NTC ) and cell number (0.008 and 0.0008) are shown. As few as two copies of pLSUO plasmid and 0.0008 cell dilutions are clearly detected. The specificity of the 204 bp amplicon was also confirmed by gel electrophoresis and sequencing analysis (data not shown).

A total of 40 qrt-PCR cycles of 10-fold serial (from 10^6^ to 10) and 5- and 2- molecule dilutions yielded a pLSUO standard curve with a dynamic range of 6 orders of magnitude and a strong linear correlation (R^2^ mean: 0.99) between the Ct values for each input amount (from 10^6^ to 2 molecules) and the log_10_ of the starting pLSUO copy number. The qrt-PCR efficiency was 95%, and the mean standard curve (*y* = −3.44x+34.04, n = 8 experiments) with a sensitivity of 2 copies/reaction was used to calculate the number of LSU gene copies per PCR sample. The gold standard curve generated by amplifying the species-specific *O.* cf. *ovata* fragment from a lysed pool of macroalgae samples (n = 4) had a linear correlation range of 5 log_10_ (R^2^ mean: 0.96), a quantification limit of 0.0008 cell per PCR reaction (Ct mean = 34.35±1.06) and an efficiency of 98%. This standard curve mean of *y* = −3.36x+23.72 (n = 8 experiments) derived from plotting the Ct values of each input amount (from 8 to 0.0008 cells) against log_10_ of the starting cell number, enabled us to calculate the initial amount of cells for each PCR sample. As the two standard curves (Fig. 1) had the same efficiency (Δs = 0.08), the data from the environmental samples were normalized and expressed as LSU gene copy number per cell.

### Assay reproducibility

The method's reproducibility (inter-assay variation) was assayed by calculating the CV_Ct_ (coefficient of variation of cycle threshold) of both pLSUO and gold standards, and for the estimated pLSUO plasmid copy number (CV_Cn_) extrapolated from the mean equation of the standard curve in 8 independent experiments run on different days using 8 different sets of dilutions for the two standard curves (Tables 1 and 2). The CV_Ct_ mean values of the pLSUO and gold standard curves were 1.7% (range 10^6^ – 2) and 3.0% (range 8 – 0.0008 cells) respectively, and remained at 1.5% in the low copy number range (10^2^ - 2 ) and at 3.0% in the low cell number range (0.008 – 0.0008). Moreover, the CV_Cn_ mean values of the pLSUO standard curve was 25% in the 10^6^ – 2 copy number range. The precision (intra-assay variation) of each of the two curves was measured 10 times within one PCR run. The mean intra-assay variation based on the Ct was 1.4% and 3.0% for the pLSUO and gold standard curves respectively.

**Table 1:** Reproducibility of the qrt-PCR assay based on pLSUO plasmid standard curve.

Copy number	Mean Ct ± SD^a^	CV_Ct_%^b^	CV_Cn_%^c^
10^6^	12.88±0.06	0.4	4.0
10^5^	16.89±0.61	3.6	33
10^4^	20.21±0.41	2.0	26
10^3^	23.68±0.38	1.6	23
10^2^	27.15±0.38	1.4	23
10^1^	30.89±0.49	1.6	33
5	31.36±0.23	0.7	15
2	32.09±0.71	2.2	46

a Mean Ct values measured in 8 independent experiments ± standard deviation (SD).

b Coefficient of variation of cycle threshold.

c Coefficient of variation of copy number.

**Table 2:** Reproducibility of the qrt-PCR assay based on gold standard curve.

Dilution factor	Cell number	Mean Ct ± SD^a^	CV_Ct_ b	CV_Ct_%
1∶1	8	20.58±0.70	0.034	3.4
1∶2	4	21.57±0.78	0.036	3.6
1∶10	0.8	24.13±0.82	0.034	3.4
1∶50	0.16	26.69±0.91	0.034	3.4
1∶100	0.08	27.52±1.12	0.041	4.1
1∶500	0.016	29.99±1.02	0.034	3.4
1∶1000	0.008	30.40±0.96	0.030	3.2
1∶2000	0.004	31.36±0.65	0.021	2.1
1∶10000	0.0008	34.35±1.06	0.031	3.1

a Mean Ct values measured in 8 independent experiments ± standard deviation (SD).

b Coefficient of variation of cycle threshold.

### Estimation of rDNA in *O.* cf. *ovata* culture system and environmental samples

Five *O.* cf. *ovata* isolates collected from different regions of the Mediterranean Sea were grown in a culture system. Aliquots of 5.0×10^3^ cells were harvested at 6, 13 and 28 days and lysated in 500 µl buffer. The qrt-PCR experiment was carried out using the crude extract derived from 20, 8, 2, and 0.2 cells per sampling day. The mean of LSU gene copy number per cell (n = 3) was calculated by plotting the C_t_ values on the pLSUO standard curve based on the dilution factor. Within the same isolate a significant variation in rDNA copy number per cell between the 6^th^ and 13^th^ days was found (*p*<0.05). On the other hand, no significant differences in copy number were detected between the 6^th^ and 28^th^ days and the 13^th^ and 28^th^ days (*p*>0.05). Meanwhile, a significant variation in copy number per cell was found among the different *O.* cf. *ovata* isolates (*p*<0.05) except for *O.* cf. *ovata* CBA166 vs CBA1377 at 13^th^ day, and CBA166 vs CBA1273, and CBA1377 vs CBA1346 at 28^th^ day (Table 3).

**Table 3:** LSU gene copy number per cell in *Ostreopsis* cf. *ovata* isolates during growth phase.

*Ostreopsis* cf. *ovata* isolates	LSU copy number ± SD^a^		
	6^th^ day	13^th^ day	28^th^ day
CBA 166	3908±188	1812±384	848±175
CBA 1273	8488±64	1372±294	3687±366
CBA 1298	1577±168	881±236	1908±300
CBA 1377	1520±155	410±54	849±210
CBA 1346	1225±217	536±121	81±13

a Mean LSU copy number measured in triplicates ± standard deviation (SD).

The LSU gene copy number per *O*. cf. *ovata* cell from environmental samples was also evaluated in qrt-PCR by extrapolating it from pLSUO and gold standard comparison curves (Tables 4 and 5). The mean value of 1030±49 derived from all both macroalga and surface seawater sample measurements, which no significant variability displayed (*p*<0.05).

**Table 4:** Qrt-PCR assay and microscopy analysis of *Ostreopsis* cf. *ovata* cell number from macroalga *Ulva rigida* samples collected in 2009 at Conero Riviera (northern Adriatic Sea) and calculation of LSU gene copy number per cell by qrt-PCR.

Sample no.	Sampling period	Abundance^a^(cells g^−1^ fw ± SD)		LSU copy no.^c^(cell^−1^ ± SD)
		qrt-PCR	Microscopy	
1	March 25^th^	n.d.^b^	n.d.	n.d.
2	April 7^th^	n.d.	n.d.	n.d.
3	May 7^th^	n.d.	n.d.	n.d.
4	May 18^th^	n.d.	n.d.	n.d.
5	June 3^rd^	n.d.	n.d.	n.d.
6	June 15^th^	n.d.	n.d.	n.d.
7	July 3^rd^	n.d.	n.d.	n.d.
8	July 17^th^	n.d.	n.d.	n.d.
9	July 31^st^	n.d.	n.d.	n.d.
10	August 6^th^	n.d.	6±8	n.d.
11	August 11^st^	n.d.	n.d.	n.d.
12	August 21^st^	7±3	n.d.	1080±63
13	August 27^th^	11±4	91±42	1150±68
14	September 3^rd^	11551±840	13838±692	972±55
15	September 9^th^	955±203	703±84	1007±62
16	September 15^th^	757±123	892±196	1030±62
17	September 23^rd^	56456±3740	55611±2224	820±41
18	September 29^th^	62120±6798	73573±2943	965±58
19	October 6^th^	28784±4056	43058±1292	960±53
20	October 21^st^	4±2	48±55	1148±68
21	October 28^th^	+	n.d.	n.d.
22	November 19^th^	n.d.	n.d.	n.d.

a Mean abundance measured in triplicates ± standard deviation (SD).

b Not detected.

c Mean LSU copy number measured in triplicates ± standard deviation (SD).

**Table 5:** Qrt-PCR assay and microscopy analysis of *Ostreopsis* cf. *ovata* cell number from surface seawater samples collected in 2009 at Conero Riviera (northern Adriatic Sea) and calculation of LSU gene copy number per cell by qrt-PCR.

Sample no.	Sampling period	Abundance^a^(cells l^−1^ ± SD)		LSU copy no.^c^(cell^−1^ ± SD)
		qrt-PCR	Microscopy	
23	March 25^th^	n.d.^b^	n.d.	n.d.
24	April 7^th^	n.d.	n.d.	n.d.
25	May 7^th^	n.d.	n.d.	n.d.
26	May 18^th^	n.d.	n.d.	n.d.
27	June 3^rd^	n.d.	n.d.	n.d.
28	June 15^th^	n.d.	n.d.	n.d.
29	July 3^rd^	n.d.	n.d.	n.d.
30	July 17^th^	n.d.	n.d.	n.d.
31	July 31^st^	n.d.	n.d.	n.d.
32	August 6^th^	n.d.	n.d.	n.d.
33	August 11^st^	n.d.	n.d.	n.d.
34	August 21^st^	n.d.	n.d.	n.d.
35	August 27^th^	11±3	160±131	1142±74
36	September 3^rd^	125924±5100	92480±3699	971±39
37	September 9^th^	100±50	360±241	1080±48
38	September 15^th^	250±75	840±370	1058±65
39	September 23^rd^	16625±775	15200±1216	1029±58
40	September 29^th^	15675±2700	13750±2062	1013±52
41	October 6^th^	2475±75	680±313	1030±40
42	October 21^st^	n.d.	20±40	n.d.
43	October 28^th^	n.d.	n.d.	n.d.

a Mean abundance measured in triplicates ± standard deviation (SD).

b Not detected.

c Mean LSU copy number measured in triplicates ± standard deviation (SD).

### Quantification of *O.* cf. *ovata* in environmental samples

All samples were analysed by qrt-PCR to check for the presence of *O.* cf. *siamensis*. These amplifications yielded negative results, excluding the presences of this species (data not shown), then all *Ostreopsis* spp. cells observed by microscopy were considered belonging to *O.* cf. *ovata*.

**Figure 2:**
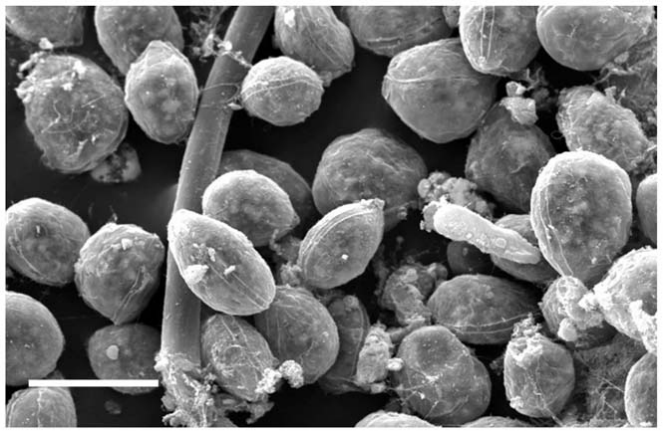
Micrograph of *Ostreopsis* cf. *ovata* assemblage in Scanning Electron Microscopy (SEM). Scale bar 50 µm (photo by Romagnoli T.).

Environmental samples of *U. rigida* microepiphytic assemblages and surface water were analysed with both microscopy and molecular (qrt-PCR assay) methods (Fig. 2). The temporal trend in *O.* cf. *ovata* mean abundances during the study period is shown in Tables 4 and 5, for epiphytic cells and water column respectively. The first occurrence of *O.* cf. *ovata* cells on macroalgal samples was detected on August 6^th^ by microscopy, and on August 21^st^ by qrt-PCR. Cell abundances increased during September and reached the peak between September 23^rd^ and October 6^th^. Maximum cell densities in the *U. rigida* samples were observed on September 29^th^ (6.2×10^4^ and 7.4×10^4^ cells g^−1^ fw by qrt-PCR and microscopy, respectively). The bloom declined at the end of October. *Ostreopsis* cells were detected until October 21^st^ by both qrt-PCR and microscopy, while on October 28^th^ a positive result was obtained only with the qrt-PCR method.

In the water column, the first cells were observed on August 27^th^ with both microscopy and qrt-PCR methods. Maximum values were observed on September 3^rd^ (1.3×10^5^ and 9.2×10^4^ cells l^−1^ detected by qrt-PCR and microscopy, respectively). As already observed with respect to the benthic substrata, cells disappeared at the end of October (last detection 20 cells l^−1^ on October 21^st^, only with microscopy). There was a significant positive correlation (n = 18, Spearman r = 0.97, *p*<0.05) between cell densities on *U. rigida* and in the water column.

A correspondence was found between the results obtained with microscopy and those found with molecular methods. This high correlation was particularly evident during the bloom event in samples from no. 14 to no. 19 and from no. 36 to no. 41 (Spearman r = 0.98 and 0.97 respectively, *p*<0.05). In the range of low cell numbers, the PCR reaction of two macrophyte samples in which no *Ostreopsis* cells were found using the volume sample settled for the microscopy, resulted in a positive detection (no. 21) and quantification of 7 cells g^−1^ fw (no. 12). Whereas, microscopy analyses revealed the presence of 6 cells g^−1^ fw in sample no. 10, and 20 cells l^−1^ in sample no. 42, while the same samples analysed by qrt-PCR produced negative results.

## Discussion

In order to understand the dynamics of toxic microalgae blooms and to mitigate their impact on human health, the environment and the economy, it is important to improve monitoring of them in terms of frequency, sensitivity and rapidity. To achieve this goal, a reliable and fast molecular method with high sample throughput and a low quantification limit is desirable. Identification of *Ostreopsis* spp. in natural samples is usually based on light or epifluorescence microscopy, but because of the high morphological and morphometrical variation within each species this is difficult, time consuming, labour-intensive, and requires a high level of taxonomic expertise, especially when different *Ostreopsis* species co-occur in natural samples.

In this study, we report the development of a qrt-PCR assay for specific *O.* cf. *ovata* quantification. The species-specific primers were designed on a partial (D1/D2 domains) LSU rDNA sequence. The qrt-PCR assay was based on binding the SYBR Green I dye into double-stranded PCR products. The SYBR Green methodology seems to be a better option for quantification than compared to sequence-specific fluorescent probes in the TaqMan or TaqMan MGB based assay, since it requires only one set of specific primers, hence providing additional experimental flexibility, and it reduces assay set-up and running costs while providing similar levels of accuracy in optimised assays [25]. Morevover, the fluorescent-probe-based assay, such as TaqMan, can fail to detect target DNA that contains even only a single mismatched base at the 5′ end of the probe nucleotide sequence. This would prevent hybridization of the 5′ end of the TaqMan probe. It seems likely that the presence of this mutation would cause no cleavage by the 5′ -3′ exonuclease activity of the *Taq* polymerase and therefore no liberation of the fluorescent reporter, making quantification impossible [26], [27].

We demonstrated that the significant variation in rDNA copy number per cell of *O.* cf. *ovata* in culture systems made it impossible to develop a reliable and accurate quantification method based only on a plasmid standard curve or only on a pool of DNA target from cultured samples. The main differences between our assay and other qrt-PCR methods were the elimination of the DNA purification step and the introduction of a second calibration curve, such as “gold standard”, which was created with pooled crude extracts of *O.* cf. *ovata* from environmental samples collected during the bloom event. This allowed us to recover total DNA of the target microbial species, eliminate the different amplification efficiencies among standards and unknown samples, and normalise the rDNA copy number per cell of *O.* cf. *ovata* in environmental samples and thus obtain a specific absolute quantification.

A series of parameters for obtaining this goal were examined. Lysis buffer performance in recovering total DNA from cells was validated by testing different samples of *O.* cf. *ovata* CBA165 collected at the same day of growth and lysed. Thus, this procedure was effective and reproducible in the range of 5000 to 50000 cells. The significant differences in LSU copy number per cell among different *O.* cf. *ovata* isolates found during growth phases is therefore due to high variability in the LSU gene content which could be due to the age of culture, as already postulated for other marine planktonic dinoflagellates [28], or to the potential presence of extra-chromosomal rDNA molecules. Indeed, in some alveolate species rRNA genes are organised in linear and circular extra-chromosomal rDNA molecules [29]. The protist *Euglena* sp. also has 800 to 4000 copies of rDNA circles per cell, depending on growth phase and culture conditions [30]. To date, no evidence has been reported supporting the presence of extra-chromosomal rDNA in *Ostreopsis* species, but this, together with the growth phases, could explain the rDNA variability obtained by qrt-PCR assay.

The mean amplification efficiency of gold standard was 98% with a 5-log linear dynamic range of quantification calculated in the low range of cells from 8 to 10^−4^ per reaction. Although the rRNA gene copy number for this species in the environmental samples is not precisely known, the fact that the quantification limit is largely below 1 cell indicates that the rDNA operon is tandemly repeated up to thousands copies, as in other protists [31]. The mean efficiency of the pLSUO standard curve was 95% with a dynamic range of 6 orders of magnitude (from 10^6^ to 2 copies) and a strong linear correlation. The similar efficiencies allowed us to precisely calculate the rDNA copy number per *O.* cf. *ovata* cell. Quantification with standards requires evaluation of precision and reproducibility in order to understand the limitations of the method. In general, a mean intra-assay variation of 10–20% and a mean inter-assay variation of 15–30% on a molecular basis (a maximum variation of 2 and 4% respectively, based on Ct) is realistic over the wide dynamic range. The data obtained from our assay confirmed the reliability and accuracy of the technical set-up over time and over the complete and the very low ranges of quantification.

Species-specificity of the assay was demonstrated: (i) *in silico* using BLAST; (ii) by qrt-PCR carried out with purified DNA from *O.* cf. *ovata* and *O.* cf. *siamensis* cultured cells; (iii) by qrt-PCR test performed with macrophyte samples containing mixed microphytobenthos assemblages to ensure the absence of non-specific amplification products. Furthermore, the results revealed that the sets of primers exclusively identified the *O.* cf. *ovata* based on the amplification of rDNA from whole cells, refuting the hypothesis that extracellular target rDNA molecules could interfere with target cell quantification. Thus, the abundance of *O.* cf. *ovata* calculated with our optimized assay was species-specific and accurate.

The qrt-PCR assay was validated in 43 environmental samples collected from the Conero Riviera during both non-bloom and bloom conditions. Cell lysates were used as templates in the PCR reactions without further purification and, to rule out the possibility that inhibitory substances affect amplification reactions, (i) the cellular pellet was washed with artificial sea water before the lysis procedure to remove traces of formalin and other inhibitors; (ii) after lysis, cellular components were eliminated by centrifugation; (iii) the efficiencies of crude extract amplifications were assayed by qrt-PCR. In addition, the spiking experiments demonstrated the absence of inhibitors. Therefore, all these devices were able to remove all PCR inhibitors from the procedure, from sample collection to PCR reaction.

Abundances determined in natural samples by qrt-PCR using the gold standard, correlated significantly with counts obtained by light microscopy during the bloom event. As far as the reliability of the microscopy method is concerned, it should be noted that in order to obtain a statistically acceptable estimate of cell numbers using the Utermöhl method, it is recommended that at least 50 units be counted for each taxon [32]. This limit is a problem for samples containing low cell abundances, such as those taken in non-bloom or pre-bloom conditions. Bearing this in mind, where cell numbers are low, data obtained through microscopic counts often overestimate cell abundances and are therefore considered highly unreliable, with an error of 200% [33]. The lack of correspondence observed between microscopic and qrt–PCR methods in environmental samples containing low cell numbers, may be explained if we take into account the threshold limits in microscopy counts. In particular, the *Ostreopsis* abundance of 20 cells l^−1^ in sample no. 42 was obtained from counting only one cell in 40 ml of settled sample, and in sample no. 10 (6 cells g^−1^ fw) only 2 cells were counted in 10 ml of settled sample. On the other hand, the sensitivity of the qrt-PCR made a positive detection in two macrophyte samples in which no *Ostreopsis* cells were found by microscopy. Furthermore, when using microscopy, sample volumes must be adjusted according to target species abundances. Concentrated samples need to be diluted, while samples with low *O.* cf. *ovata* abundances need to be counted in higher volumes or even in multiple sub-samples. These complications mean that it could be take as long as two weeks to fully process the analyses of the 43 samples. In contrast, with our qrt-PCR method it takes few hours to analyse a set of standards and unknown samples, reducing working time drastically compared with microscopy-based methods when a large number of samples need to be analysed. The qrt-PCR we developed had a high specificity, sensitivity, reproducibility and efficiency in a broad dynamic range over which cell abundance could be quantified, and did not require morphological taxonomic expertise. This approach involved the analysis of 40-ml field samples and resulted in a quantification limit based on both sample and lysis buffer volumes. This quantification limit could be reduced by filtering a larger sample volume, which would increase the sensitivity of the qrt-PCR assay.

The similar efficiencies of the pLSUO and gold standard curves that we found allowed us to correctly quantify the mean copy number of rDNA per cell (1030±49) in the *O.* cf. *ovata* bloom event. This is very important because for the first time a molecular assay that was validated on *O.* cf. *ovata* quantification by pLSUO and gold standards has allowed us to quantify the toxic benthic dinoflagellate in a survey activity using only the pLSUO standard curve. To demonstrate the robustness of the qrt-PCR method we applied it to new samples collected during the *O.* cf. *ovata* bloom occurred at the Conero Riviera in 2010 summer. The comparison of results obtained from these preliminary experiments with those obtained in the present study, showed high reproducibility and efficiency of the method at time scale. Therefore, the assay may be considered versatile to any environmental bloom in different Mediterranean coastal localities.

Furthermore, the timely and specific detection of harmful algal species prior to bloom development is a crucial component of most HAB management programmes and is also a necessary tool for researchers studying population dynamics and developing models to forecast HAB events.
